# Keys to Cultivating Uncultured Microbes: Elaborate Enrichment Strategies and Resuscitation of Dormant Cells

**DOI:** 10.1264/jsme2.ME3004rh

**Published:** 2015-12

**Authors:** Yoichi Kamagata

**Affiliations:** 1 Bioproduction Research Institute, National Institute of Advanced Industrial Science and Technology (AIST)Tsukuba, Central 6, Tsukuba, Ibaraki 305–8566Japan

Over the last few years, extensive investigations have been performed in an attempt to isolate yet-to-be cultivated microorganisms that are of ecological importance (1, 4, 12, 19). The enrichment, isolation, characterization, and genome sequencing of these organisms are classical, but robust processes that explicitly unveil their entity (5, 6, 9). However, difficulties are associated with the direct isolation of target microbes, particularly previously uncultured microbes. Enrichment is a long-used and common practice, and effectively increases the population of target organism(s), whereas bias caused by enrichment conditions always has to be taken into consideration. The purposes of the enrichment of specific microbes or communities are two-fold. One purpose is to enrich organisms that metabolize/degrade/consume a target compound. The compound may be, for example, a hazardous chemical, oil, or organic polymer. It may also be the oxidized form of a metal, sulfate, or nitrate as an electron acceptor, or the reduced form of a metal, sulfide, fatty acid, or sugar as an electron donor. The other purpose is to enrich organisms that produce metabolites such as antibiotics, polymeric compounds, and extracellular enzymes, most of which are of industrial importance.

Enrichment in which polyhydroxyalkanoate-accumulating organisms (PHAAOs) dominate was successfully constructed by Oshiki *et al.* (13). They collected activated sludge samples from 8 full-scale wastewater treatment plants operated in the fully aerobic mode. Activated sludge samples were then incubated with the addition of acetate, which allowed PHAAOs to accumulate PHAs. By employing this approach, PHAAOs were highly enriched, accounting for 11 to 18% of all cells.

Fujitani *et al.* performed the selective enrichment of two *Nitrospira*-like nitrite-oxidizing bacteria from a wastewater treatment plant. Nitrite oxidizers are well-known microorganisms that escape cultivation. The authors strictly controlled nitrite concentrations in the bioreactor to less than 10 mg N L^−1^ over two years of operation, resulting in the selective enrichment of sublineages I and II of the genus *Nitrospira*, which play a key role in nitrite oxidation in wastewater plants. The maximum ratios of sublineages I and II to all microbial cells were 88.3% and 53.8%, respectively. This study also showed that the two sublineages of uncultured *Nitrospira* were sensitive to high concentrations of nitrite with high affinity for the substrate. This enrichment method is very effective for uncultured nitrite oxidizers (2). This group eventually isolated a bacterium belonging to sublineage II of the genus *Nitrospira* (20) using a cell-sorting system.

Iino *et al.* obtained a highly enriched methanogen that was phylogenetically novel. *Candidatus* Methanogranum caenicola within the class *Thermoplasmata* was enriched from digested sludge. Although the media used were insignificant, methanol with a trace amount of yeast extract as energy and nutrient sources led to the successful enrichment of the novel methanogen (3).

Oshiki attempted to cultivate the planktonic anaerobic ammonium-oxidizing (Anammox) bacteria *Ca.* Brocadia sinica and *Ca.* Scalindua sp. in the form of planktonic cells using membrane bioreactors (MBRs). MBRs were continuously operated for more than 250 d with two different nitrogen-loading rates. *Ca.* Brocadia sinica and *Ca.* Scalindua sp. cells were successfully enriched (>90%) in MBRs with high and very low nitrogen-loading rates, respectively, as revealed by fluorescence *in situ* hybridization and a 16S rRNA gene sequencing analysis. This study demonstrated that MBRs are very effective at highly enriching planktonic anammox bacterial cells because the membrane prevents free living cells from washout (14).

Benzene is a well-known recalcitrant aromatic compound under methanogenic conditions; therefore, the enrichment and isolation of benzene-degrading organisms are challenging. Noguchi *et al.* previously attempted to enrich benzene-degrading methanogenic communities (11). In more than three years of a batch culture with benzene as the sole substrate, they obtained a community capable of degrading 98% of 1 mM benzene. ^13^C-labeled benzene combined with terminal restriction fragment length polymorphism (T-RFLP) profiles and a 16S rRNA gene cloning analysis of the buoyant density fractions revealed the incorporation of ^13^C into two phylotypes: *Desulfobacterales*- and *Coriobacteriaceae*-related bacteria. However, the enrichment constructed still harbored a diverse array of organisms, thereby highlighting the difficulties associated with the simplification of community members for such a persistent chemical. This may be attributed to many kinds of microorganisms being required for degradation and community stability.

Narihiro *et al.* attempted to demonstrate how enrichment causes bias by comparing direct isolation from a natural environmental sample together with a metanogenomic analysis (10). Comprehensive approaches were employed including functional metagenomics and a cultivation-dependent method combined with oil-fed enrichment in order to uncover the entire microbial resources of lipolytic enzymes (lipases). The findings obtained showed that isolates from oil-fed enrichments differed from those from a natural sample; however, most were organisms known to be lipase-producing bacteria. Moreover, they found that metagenomic clones encoding lipases associated with *Alphaproteobacteria*, *Deltaproteobacteria*, *Acidobacteria*, *Armatimonadetes*, and *Planctomycetes* and hitherto-uncultivated microbes were recovered not only from a non-enriched natural sample, but also from oil-fed enrichment. Collectively, these findings strongly indicate that although enrichment selects particular organisms, the lipase-producing species obtained from enrichment and natural samples are not novel, whereas functional metanogenomics effectively captures as yet undiscovered lipases that have eluded cultivation.

An important point to consider in the isolation of microbes is that most environmental microbes are difficult to cultivate. However, an important question remains unanswered: are yet-to-be cultivated organisms alive or dormant? It currently remains unknown whether the difficulties associated with growing environmental organisms due to their physiological states hinder cultivation. Microorganisms have two basic physiological states: alive and dead (15). However, as reported in previous studies on *Mycobacterium tuberculosis*, *Micrococcus luteus*, and *Rhodococcus rhodochrous*, some microbes enter a dormant stage, which is an alive, but non-growing stage. Resuscitation-promoting factors (RPFs) contribute to the “waking up” of dormant cells (8, 18). Due to their effects at very low concentrations on resuscitation, they are called bacteriocytokines. Recent studies showed that not only these bacteria, but also other organisms within the phylum *Actinobacteria* encode rpf genes, and the RPF protein was found to be functional (16, 17). Resuscitation by pyruvate was also reported in *Salmonella*, one of the most frequent causative agents for food-borne diseases (7). *Salmonella* is known to persist and survive in the environment even after exposure to sanitizers such as hydrogen peroxide (H_2_O_2_). Morishige *et al.* found that H_2_O_2_ exposure rapidly compelled *Salmonella enterica* serovar Enteritidis (SE) cells to enter the dormant stage. They also showed that pyruvate and its analog, α-ketobutyrate, had resuscitative effects on H_2_O_2_-treated SE cells in a dose-dependent manner.

Dormancy has long been discussed since the first finding referred to as viable but not culturable (VBNC) (21). Studies are currently being performed in order to obtain a better understanding on the physiological states of microorganisms in natural environments, and may also provide insights into the cultivation of microbes, *i.e.* strategies to improve the cultivation of yet-to-be cultured organisms.
